# Effectiveness of prophylactic retropharyngeal lymph node irradiation in patients with locally advanced head and neck cancer

**DOI:** 10.1186/1471-2407-12-253

**Published:** 2012-06-18

**Authors:** Nam P Nguyen, Jacqueline Vock, Vincent Vinh-Hung, Fabio Almeida, Lars Ewell, Michael Betz, Siyoung Jang, Richard A Vo, Suresh Dutta, Juan Godinez, Ulf Karlsson, Alexander Chi

**Affiliations:** 1 Department of Radiation Oncology, University of Arizona, Tucson, AZ, USA; 2 Department of Radiation Oncology, University of Bern, Bern, Switzerland, NC, USA; 3 Department of Radiation Oncology, University Hospitals of Geneva, Switzerland, Geneva, USA; 4 University of Galveston Medical School, Galveston, TX, USA; 5 San Antonio Cancer Center, San Antonio, TX, USA; 6 Florida Radiation Oncology Group, Palatka, FL, USA; 7 Marshfield Clinic, Marshfield, WI, USA; 8 Radiation Oncology, University of Arizona, 1501 N. Campbell Ave, Tucson, AZ 85724-5081, USA; 9 Department of Radiation Oncology, Southwest PET/CT Institute, Tucson, AZ, USA

**Keywords:** Head and neck cancer, IMRT, IGRT, Retropharyngeal lymph nodes

## Abstract

**Background:**

The aim of the study is to assess the effectiveness of intensity-modulated radiotherapy (IMRT) or image-guided radiotherapy (IGRT) for the prevention of retropharyngeal nodal recurrences in locally advanced head and neck cancer.

**Methods:**

A retrospective review of 76 patients with head and neck cancer undergoing concurrent chemoradiation or postoperative radiotherapy with IMRT or IGRT who were at risk for retropharyngeal nodal recurrences because of anatomic site (hypopharynx, nasopharynx, oropharynx) and/or the presence of nodal metastases was undertaken.

The prevalence of retropharyngeal nodal recurrences was assessed on follow-up positron emission tomography (PET)-CT scans.

**Results:**

At a median follow-up of 22 months (4–53 months), no patient developed retropharyngeal nodal recurrences.

**Conclusion:**

Prophylactic irradiation of retropharyngeal lymph nodes with IMRT or IGRT provides effective regional control for individuals at risk for recurrence in these nodes.

## Background

Intensity-modulated radiotherapy (IMRT) and more recently image-guided radiotherapy (IGRT) have largely replaced conventional radiotherapy in the treatment of head and neck cancer, thanks to their superior ability to spare normal tissues at risk for complications such as the parotid glands, larynx, and pharynx [1-3]. There is however, a potential flipside in that target areas that are routinely irradiated with conventional radiotherapy, might be overlooked with IMRT. One particular important clinical target volume in head and neck cancer is the retropharyngeal (RP) lymph nodes.

Anatomically the RP is the space behind the upper part of the pharynx and in front of the arch of the atlas in the buccopharyngeal fascia. The RP is important in advanced head and neck cancer because it is a site of direct extension and lymph node metastases, but surgical treatment is limited, and a high rate of involvement has been reported in diagnosic imaging studies. Delineation the RP was not a concern in conventional radiotherapy, since treatment is performed by large lateral fields that encompass the whole neck, and posterior border of boost fields are typically at mid vertebra, therefore RP lymph nodes are included, whether or not attention is given to the RP. With IMRT, delineation of the RP is explicitly required in order to include it in the treatment volumes. At the University of Arizona, indeed the standard is to include the RP for prophylactic irradiation by IMRT and IGRT in advanced head and neck cancer. We evaluate the outcome of this treatment strategy in this retrospective study.

Indeed, the true prevalence of retropharyngeal lymph node metastases is not known with certainty, given that these nodes are neither accessible to clinical examination nor routinely dissected during a radical or modified radical neck dissection. In selected studies of hypopharyngeal cancers where RP nodal dissections were performed, up to 20% of the lymph nodes contained metastatic deposits [4,5]. For non-invasive detection of RP nodal metastases, while magnetic resonance imaging (MRI) has been shown to be preferred to computed tomography (CT) scans, positron emission tomography (PET)-CT scan is emerging as diagnostic study of choice for the staging of head and neck cancer [6,7]. Preliminary studies suggest that the accuracy of PET-CT imaging in diagnosing RP lymph nodes metastases may be superior to MRI [8,9]. In two recent studies, the prevalence of RP nodal metastases ranged from 17-20% for oro-, and hypo-pharyngeal cancer, and up to 86% for nasopharyngeal cancer by MRI or PET-CT scan [10,11]. As patients with RP nodal metastases usually have poorer survival rates compared to those without them [12-16], preventing RP nodal recurrence is an important issue for radiotherapy planning in patients who are at a high risk of such recurrence.

## Methods

The medical records of 76 patients undergoing radiotherapy for locally advanced head and neck cancers at the University of Arizona, Department of Radiation Oncology, were retrospectively reviewed following institutional review board (IRB) approval. One patient consented for his clinical imaging to be published following de-identification. Patients were selected if they had naso-, oro-, and hypopharyngeal cancers and/or regional nodal metastases from head and neck primary tumors of any site.

All patients had PET-CT scans prior to treatment for staging purposes. Patients who had retropharyngeal lymph nodal metastases on the pre-treatment PET-CT were excluded. Head and neck MRI was not performed routinely except for patients with nasopharyngeal cancer because of its superiority in the detection of intracranial extension. All patients were treated with the whole field IMRT (Elekta) or IGRT (Helical Tomotherapy, Hi-Art II) with simultaneous integrated boost, from January 2006 to May 2011. Prior to treatment, each patient was simulated in the supine position with a head and neck aquaplast mask for treatment immobilization. A computed tomography (CT) scan with and without intravenous (IV) contrast for treatment planning was performed in the treatment position. The head and neck areas from the vertex to the mid thorax were scanned with a slice thickness of 3 mm CT scan with IV contrast to outline the tumor and grossly enlarged cervical lymph nodes for target volume delineation. Radiotherapy planning was performed on the CT scan without contrast to avoid possible interference of contrast density on radiotherapy isodose distributions. Diagnostic PET-CT scan planning for tumor imaging was also incorporated with CT planning for tumor imaging. A 0.5 cm bolus material was placed on any area of the skin involved by the tumor and on any palpable cervical lymph nodes. Normal organs at risk for complication were outlined for treatment planning (spinal cord, brain stem, bilateral cochlea, mandible, parotid glands, bilateral eyes, and oral cavity). For patients with definitive chemoradiation, the tumor and grossly enlarged lymph nodes (CTV1) on CT scan with a margin (PTV1) were treated to 70 Gy in 35 fractions (2 Gy/fraction). The margins were 5 mm to 1 cm all around CTV1 depending on anatomic location. The high risk-PTV2 (at least 1 cm around gross tumor and pathologic cervical lymph nodes) and low risk -PTV3 (subclinical regional lymph nodes with 5 mm margins) for tumor spread were treated respectively to 63 Gy and 56 Gy in 35 fractions, respectively. Patients undergoing postoperative radiation or chemoradiation were treated to 66 Gy, 59.4 Gy, and 54 Gy in 33 fractions to PTV1, PTV2, and PTV3 respectively. Indications for postoperative chemoradiation were positive margins and/or lymph node metastases with extra-capsular extension. Minimal target coverage was 95% of the prescribed dose for all targets with at least 99% of the prescribed dose delivered to gross tumor and involved cervical lymph nodes. The lymph nodes in the ipsilateral neck including the retropharyngeal lymph nodes were treated to the base of skull if there was any cervical lymph node enlargement (or PET-positive lymph nodes). The retropharyngeal lymph nodes were outlined from the hyoid bone to the base of skull to include the Rouviere nodes based on the consensus guidelines [17]. The retropharyngeal lymph nodes were treated to 5600 cGy (35 fractions) during exclusive chemoradiation, or 5400 cGy (33 fractions) during postoperative radiation or chemoradiation because of the risk for subclinical disease. Contralateral uninvolved lymph nodes were treated prophylactically with the C1 vertebrae as the superior border of the radiation field in order to spare the parotid gland. In case of bilateral regional metastases, both sides of the neck were irradiated to the base of skull to avoid any marginal miss. Mean dose to the parotid gland was constrained below 2600 cGy if there was no ipsilateral cervical lymph node enlargement. Dose constraints for other normal organs at risk (OAR) for complications were: Dmax to the spinal cord <45 Gy, Dmax to the brain stem <50 Gy, Dmax to the optic chiasm <45 Gy, and mandibular V70 <30% of the mandible.

Concurrent chemoradiation was recommended for all except for patients with thyroid malignancies (2). One patient with neuroendocrine tumor of the thyroid was treated with concurrent chemoradiation The type of chemotherapy regimen was left to the discretion of the medical oncologists depending on patient functional status and co-morbidities. Prophylactic percutaneous gastrostomy tubes feedings placement was recommended for all patients prior to radiotherapy because of the expected weight loss secondary to severe mucositis, dysphagia, and dysgueusia. Weekly complete blood count (CBC) and blood chemistry to assess renal function were performed during chemoradiation. Treatment breaks and weight loss were recorded during chemoradiation.

Acute and long-term toxicities were graded according to Radiotherapy Oncology Group (RTOG) group criteria (http://ctep.cancer.gov).

All patients had a follow-up visit at one month, then regularly every three months following treatment. Clinical and direct endoscopic examination were performed at each follow-up visit to detect recurrent disease. A PET-CT scan was performed at four months and ten months, then yearly after treatment if there was no evidence of disease. PET-positive areas were biopsied to detect local recurrences and surgery and/or chemotherapy were carried out for salvage if the biopsy demonstrated malignancy. The PET-CT scans following radiotherapy were reviewed, compared to the pre-treatment PET-CT and discussed with the radiologist to assess for recurrent disease and specifically, the presence or absence of retropharyngeal lymph node metastases.

## Results

We identified 76 patients with locally advanced head and neck cancer treated at the University of Arizona, Department of Radiation Oncology from 2006 to 2011. Median age at diagnosis was 59 years-old (range: 25–84 years-old). There were 66 males and 10 females. There were 21 stage III, 32 stage IVA, 18 stage IVB, 2 stage IVC, and three recurrences. Seventy-four patients had nodal metastases. Treatment consisted of: postoperative radiation (2), postoperative chemoradiation (16), and definitive concurrent chemoradiation (58). Seven patients were treated with IMRT and the other 69 patients were treated with IGRT.

Table 1 summarizes patient characteristics. At a median follow-up of 22 months (4–53 months), no patient developed any recurrences in the retropharyngeal lymph nodes. Local recurrence developed in 8 patients (10%). One patient who had local recurrence also developed regional recurrence and distant metastases. No other patient developed regional recurrence. Ten patients (13%) developed distant metastases (7 lungs, 2 bones, and 1 brain). Four patients (5%) developed second primaries (2 lungs, 1 bladder, and 1 prostate). The two patients with second lung primaries were salvaged with surgery (1) and stereotactic body radiotherapy (1). The other two patients died from their second primaries. Overall 65 patients (85%) were alive at the last follow-up.

**Table 1:** Patient characteristics

**Patient No.**	**76**	
Age		
Median	59	
Range	25-84	
Sex		
Male	66	
Female	10	
Histology		
Squamous	72	
Adenocarcoma	2	
Neuroendocrine	2	
Sites		
Oropharynx	42	
Larynx	12	
Oral cavity	6	
Hypopharynx	4	
Unknown	3	
Thyroid	3	
Nasopharynx	4	
Maxillary sinus	2	
Stages		
III	21	
IVA	32	
IVB	18	
IVC	2	
Recurrence	3	
T stages		
Tx	3	
T1	7	
T2	28	
T3	13	
T4	22	
Recurrence	3	
Neck nodes		
N0	2	
N1	29	
N2	36	
N3	9	
Unilateral	55	
Bilateral	19	
Treatement		
Post operative radiotherapy	2	
Postoperative chemoradiation	16	
Definitive chemoradiation	58	
Follow-up (months)		
Median	22	
Range	4-53	

## Discussion

To our knowledge, this is the first study looking at the effectiveness of IMRT and IGRT in preventing of retropharyngeal nodal recurrence in the setting of locally advanced head and neck cancer using PET scan for staging, planning, and follow-up.

**Figure 1:**
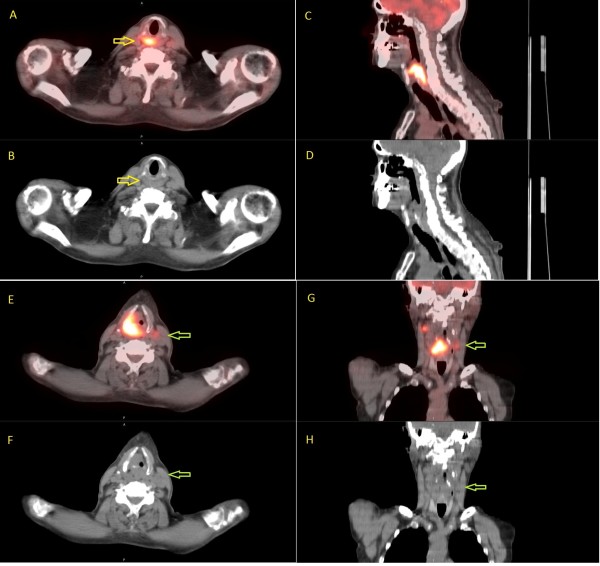
**PET-CT scan of a patient with locally advanced laryngeal cancer illustrating the importance of this imaging technique for radiotherapy treatment planning.** The tumor invades the cricoid cartilage inferiorly in addition to the contralateral neck nodes which was not observed on the pre-treatment diagnostic CT scan. The gross tumor and the contralateral neck nodes would have been underdosed without the planning PET-CT scan. On the other hand, despite the presence of bilateral cervical lymph nodes, no retropharyngeal (RP) lymph node metastases was observed. Thus, regional control may be achieved with a prophylactic radiation dose (56 Gy) to the RP area while a curative dose (70 Gy) is required for the PET positive gross tumor and lymph nodes. Patients with supraglottic laryngeal cancer and cervical lymph nodes metastases may be at higher risk for RP nodal failure. The patient is free of disease eight months following treatment. Axial fused PET/CT (**A**) shows extent of metabolic activity inferior to the cricoid cartilage [yellow arrow] which is not anatomically visible on the CT alone (**B**). Sagittal PET/CT (**C**) and CT (**D**) showing cranial-caudal extent of tumor. Axial and Coronal PET/CT (**E**&**G**) showing a metabolic contralateral node [green arrow], also difficult to appreciate on the CT alone (**F**&**H**).

Prophylactic RP nodes irradiation may improve survival in patients who are at a high risk for RP nodal metastases, the occurrence of which has been demonstrated in many studies to be an independent prognostic factor for survival. In a study of 208 oropharyngeal cancer patients, the regional recurrence rates were 45% and 10% respectively, in the presence or absence of positive RP nodes on diagnostic CT scans, while the 5-year disease-specific survival rates were 38% and 58% [14]. In another study of 366 hypophagyngeal cancer patients treated with surgery including RP nodal dissection, 54 patients (14.8%) had positive RP nodes. In 41 of these patients had long-term follow-up. Local recurrences developed in 21 patients and distant metastases occurred in another 8 [18]. Mc Laughlin et al also corroborated the poor survival and high recurrence rate related to RP adenopathy diagnosed on CT scans and MRI in 619 patients of various anatomic sites [15]. In head and neck cancer patients at high risk for subclinical RP nodal involvement because of anatomic sites and/or cervical lymph nodes metastases, prophylactic RP nodal irradiation may potentially decrease the loco-regional recurrence rate, while lack of coverage of the RP nodes may lead to recurrence and poor survival. Indeed, in one study of 133 non-nasopharyngeal cancer patients treated with IMRT, RP nodes recurrence occurred in three patients because of marginal miss [19]. In contrast to our study, PET-CT scans were not employed for staging and radiotherapy planning in that study, thus raising the question that these three patients may already have had RP nodes metastases which were not diagnosed because of the low sensitivity of diagnostic CT scans. For example, in a study of 105 nasopharyngeal carcinoma comparing the accuracy of PET-CT and MRI for cervical lymph node staging, 30 cervical nodes were positive on PET-CT but negative on MRI [9]. Long-term follow-up demonstrated recurrence in 25 patients. Our study, by including PET-CT imaging in the staging of all patients, none of whom had positive RP nodes on PET-CT, is potentially the first to demonstrate the value of prophylactic RP nodal irradiation. Our study also emphasizes the need to incorporate PET-CT scans into radiotherapy planning and for follow-up because of its high accuracy to delineate the tumor extent and regional lymph nodes metastases. Despite the fact that almost all patients had cervical nodal metastases before treatment (one patient with T4 nasopharyngeal cancer did not have nodal metastases and the other one had a recurrence of base of tongue cancer), only one patient developed regional recurrence following radiotherapy. In addition, four patients developed second primaries after treatment observed on PET-CT scans allowing successful salvage therapy in two patients. As a result, the success of new radiotherapy treatment modalities such as IGRT will be dependent on our ability to delineate the gross tumor and regional lymph nodes accurately in order to allow radiation dose escalation and for normal tissues sparing. Figure 1 demonstrates a supraglottic cancer which invaded the cricoid cartilage inferiorly and involved the contralateral cervical lymph node on the planning PET-CT scan. The diagnostic CT scan did not show these features and would have led to loco-regional recurrence if PET-CT was not performed for treatment planning. On the other hand, despite the presence of bilateral cervical lymph nodes, no RP lymph nodes metastases were observed, thus allowing us to treat the RP area with a prophylactic dose of radiation (56 Gy) while treating the gross tumor and cervical nodes to a curative dose (70 Gy). Patients with supraglottic laryngeal cancers and cervical lymph nodes metastases may be at higher risk for RP nodal failure [15]. The incorporation of PET-CT into radiotherapy planning may have accounted for improved regional control as only one patient developed regional failure despite the fact that most of the patients had cervical lymph nodes metastases at diagnosis. We also included patients with a relatively lower rate of RP nodal recurrence from other anatomic sites such as the larynx, oral cavity, and thyroid because of the presence of neck nodes metastases. Patients with thyroid cancer may develop RP nodal metastases following surgery because of alteration of the lymph node drainage in a retrograde fashion [20-23]. Although less common, RP recurrences have been also reported in other anatomic sites [24,25]. Compared to those sites, maxillary sinus cancers had a relatively higher rate of RP nodal failure [26]. As there is no consensus on the regional lymph nodes at risk for recurrence in the presence of neck nodes metastases, we elected to treat the RP nodal area to prophylactic dose of radiation as those patients may have a higher rate of RP metastases compared to patients with no neck nodes [15].

The limitations of the present study include the retrospective nature of the study, the small number of patients, and the short follow-up. However, our study demonstrates that with proper PET-CT staging, prophylactic RP nodal irradiation may decrease the recurrence rate in patients at risk for regional failure. Further prospective studies with a large number of patients and long-term follow-up are required to confirm our hypothesis.

## Conclusion

Prophylactic retropharyngeal lymph nodes irradiation with IMRT or IGRT may decrease the incidence of retropharyngeal recurrences in patients at risk for RP nodal metastases. Incorporating PET-CT into radiotherapy treatment planning may improve loco-regional control.

## Conflict of interest

The authors have no conflict of interest and have no source of funding.

## Authors’ contribution

NPN, LE, and AC collected the data. All authors participated in the study design, data interpretation, and writing of draft. All authors read and approved the manuscript.

## Pre-publication history

The pre-publication history for this paper can be accessed here:

http://www.biomedcentral.com/1471-2407/12/253/prepub

## References

[B1] Kam MK Leung SF Zee B Chau RM Suen JJ Mo F Lai M Ho R Cheung KY Yu BK Chiu SK Choi PH Teo PM Kwan WH Chan AT Prospective randomized study of intensity-modulated radiotherapy on salivary gland function in early-stage nasopharyngeal carcinoma patients J Clin Oncol 2007 25 4873 4879 10.1200/JCO.2007.11.5501 17971582

[B2] Nguyen NP Ceizyk M Vos P Vinh-Hung V Davis R Desai A Abraham D Krafft SP Jang S Watchman CJ Ewell L Hamilton R Smith LR Effectiveness of image-guided radiotherapy for laryngeal sparing in head and neck cancer Oral Oncol 2010 46 283 286 10.1016/j.oraloncology.2010.01.010 20188620

[B3] Nguyen N Smith-Raymond L Vinh-Hung V Sloan D Davis R Vos P Abraham D Stevie M Krafft SP Ly BH Ries T Karlsson U Ceizyk M Feasibility of Tomotherapy to spare the cochlea from excessive radiation in head and neck cancer Oral Oncol 2011 47 414 419 10.1016/j.oraloncology.2011.03.011 21474364

[B4] Okumura K Fujimoto Y Hasegawa Y Matsuura H Nakayama B Komura T Ogawa T Terada A Matsuzuka T Retropharyngeal node metastasis in cancer of the oropharynx and hypopharynx: analysis of retropharyngeal node dissection regarding preoperative radiographic diagnosis Nippon Jibiinkoka Gakkai Kaiho 1998 101 573 577 10.3950/jibiinkoka.101.5_573 9642997

[B5] Amatsu M Mohri M Kinishi M Significance of retropharyngeal node dissection at radical surgery for carcinoma of the hyppharynx and cervical esophagus Laryngoscope 2001 111 1099 1103 10.1097/00005537-200106000-00031 11404628

[B6] Liu LZ Zhang GY Xie CM Cui CY Li L Magnetic resonance imaging of retropharyngeal lymph node metastasis in nasopharyngeal carcinoma Int. J. Radiat Oncol Bio Phys 2006 66 721 730 10.1016/j.ijrobp.2006.05.054 17011448

[B7] Tomita N Fuwa N Ariji Y Kodaira T Mizoguchi N Factors associated with nodal metástasis in nasopharyngeal cáncer: an approach to reduce the radiation field in selected patients Br J Radiol 2011 84 265 270 10.1259/bjr/47164832 20959372 PMC3473866

[B8] Chu HR Kim JH Yoon DY Hwang HS Rho YS Additional diagnostic value of (18)F-FDG PET-CT in detecting retropharyngeal lymph node metastases Otolaryngol Head Neck Surg 2009 141 633 638 10.1016/j.otohns.2009.08.008 19861203

[B9] Hu WH Zhang GY Liu LZ Wu HB Li L Gao YH Pan Y Wang QS Comparison between PET-CT and MRI in diagnosing nodal metastasis of nasopharyngeal carcinoma Chinese J Cancer 2005 24 855 860 16004815

[B10] Chan SC Lin CY Ng SH Chang JT Wang HM Liao CT Lo CW Yen TC 18F-FDG PET for lymph node metastasis in oropharyngeal and hypopharyngeal cancers: impact on diagnosis and prediction analysis Nucl Med Comm 2010 31 260 265 10.1097/MNM.0b013e3283360133 20072075

[B11] Tauzin M Rabalais A Hagan JL Wood CG Ferris RL Walvekar RR PET-CT staging of the neck in cancers of the oropharynx: patterns of regional and retropharyngeal nodal metastasis World J Surg Oncol 2010 8 70 10.1186/1477-7819-8-70 20712875 PMC2929233

[B12] Tang L Mao Y Liu L Chen Y Sun Y Liao X Lin A Liu M Li L Ma J The volume to be irradiated during selective head and neck irradiation in nasopharyngeal cancer Cancer 2009 115 680 688 10.1002/cncr.24049 19117352

[B13] Tang L Li L Mao Y Liang S Chen Y Sun Y Liao X Tian L Lin A Liu M Ma J Retropharyngeal lymph nodes metastasis in nasopharyngeal carcinoma detected by magnetic resonance imaging Prognostic value and staging categories. Cancer 2008 113 347 354 10.1002/cncr.23555 18459174

[B14] Dirix P Nuyts S Bussels B Hermans R Van den Bogaert W Prognostic influence of retropharyngeal lymph node metastasis in squamous cell carcinoma of the oropharynx Int J Radiat Oncol Phys 2006 65 739 744 10.1016/j.ijrobp.2006.02.027 16751062

[B15] McLaughlin MP Mendenhall WM Mancuso AA Parsons JT McCarthy PJ Cassisi NJ Stringer SP Tart RP Mukherji SK Million RR Retropharyngeal adenopathy as a predictor of outcome in squamous cell carcinoma of the head and neck Head Neck 1995 17 190 198 10.1002/hed.2880170304 7782203

[B16] Ogino I Tsukuda M Inoue T Mikami Y Itazawa T Matsuda H Koiki I Horiuchi C Omura M Taguchi TL Prognostic value of retropharyngeal node involvement in CT-based lymph node positive pharyngeal cancer following radiotherapy Anticancer Res 2007 27 1663 1668 17595793

[B17] Gregoire V Levendag P Ang KK Bernier J Braaksma M Budach V Chao C Coche E Cooper JS Cosnard G Eisbruch A El-Sayed S Emami B Grau C Hamoir M Lee N Maingon P Muller K Reychler H CT-based delineation of lymph node levels and related CTVs in the node negative neck: DAHANCA, EORTC, GORTEC, NCIC, RTOG consensus guidelines Radiother Oncol 2003 69 227 236 10.1016/j.radonc.2003.09.011 14644481

[B18] Yoshimoto S Kawabata K Retropharyngeal node dissection during total pharyngolaryngectomy for hypopharyngeal cancer Auris Nasus Larynx 2005 32 163 167 10.1016/j.anl.2004.11.003 15917174

[B19] Eisbruch A Marsh LH Dawson LA Bradford CR Teknos TN Chepeha DB Worden FP Urba S Lin A Schipper MJ Wolf GT Recurrences near base of skull after IMRT for head and neck cancer: Implications for target delineation in high neck and for parotid gland sparing Int J Radiat Oncol Biol Phys 2004 50 28 42 15093896 10.1016/j.ijrobp.2003.10.032

[B20] Otsuki N Nishikawa T Iwae S Saito M Mohri M Nibu K Retropharyngeal node metastasis from papillary thyroid carcinoma Head Neck 2007 29 508 511 10.1002/hed.20536 17120310

[B21] Andrews GA Kwon M Clayman G Edeiken B Kupferman ME Technical refinement of ultrasound-guided transoral resection of parapharyngeal/retropharyngeal thyroid carcinoma metastases Head Neck 2011 33 166 170 10.1002/hed.21415 20848435

[B22] Aygenc E Kaymakci M Karaka C Ozdem C Papillary thyroid carcinoma metastasis to the parapharyngeal space Eur Arch Otorhinolaryngol 2002 259 322 324 12115081 10.1007/s00405-002-0466-x

[B23] Shellenberger T Fornage B Ginsberg L Clayman Gl Transoral resection of thyroid cancer metastasis to the lateral retropharyngeal nodes Head Neck 2007 29 258 266 10.1002/hed.20513 17022089

[B24] Umeda M Shigeta T Takahashi H Kataoka T Oguni A Minamikawa T Shibuya Y Yokoo S Komori T Metastasis to the lateral retropharyngeal Lymph node from squamous cell carcinoma of the oral cavity Int J Oral Maxillofacial Surg 2009 38 1004 1008 10.1016/j.ijom.2009.04.015 19467843

[B25] Seniukov MV Laryngeal cancer metastasis into the retropharyngeal lymph nodes Vestn Otorinolaringol 5 51 54 1081 7303389

[B26] Watarai J Seino Y Kobayashi M Shindo M Kato T CT of retropharyngeal lymph node metastasis in head and neck squamous cell carcinoma Acta Radiol 1993 34 492 495 10.3109/02841859309175391 8396404

